# Improved affinity AFP-specific T cell receptor for hepatocellular carcinoma

**DOI:** 10.1186/2051-1426-1-S1-P10

**Published:** 2013-11-07

**Authors:** Andrew B Gerry, Nicholas J Pumphrey, Roslin Y Docta, Joanna E Brewer, Alan D Bennett, Bent K Jakobsen

**Affiliations:** 1 Adaptimmune Ltd, Abingdon, UK

## 

We have previously demonstrated that an improved affinity TCR generated against NYESO-1 has shown remarkable efficacy in clinical studies in multiple myeloma (NCT01352286). The same TCR is also being tested in synovial sarcoma (NCT01343043), melanoma (NCT01350401) and ovarian (NCT01567891) clinical trials. We will extend these clinical studies to include hepatocellular carcinoma shortly. In this present study, we have generated a panel of improved-affinity TCRs for a widely expressed hepatic carcinoma-specific HLA-A2-restricted peptide antigen AFP158-166. This TCR panel demonstrates stepwise increases in affinity from wildtype to approximately 500-fold greater binding to cognate antigen. These affinity-enhanced TCRs were screened for efficacy and specificity by a variety of in vitro assays, such as interferon gamma ELISpot, Incucyte™ cytotoxicity assays for visualizing caspase 3/7 activity, Luminex™ multiple cytokine secretion assays and 3D microtissue models against a variety of hepatocellular carcinoma cell lines and normal primary cell types. This TCR panel demonstrated a window of activity whereby moderately increased affinity TCRs showed maximal response to antigen positive hepatocellular carcinoma cell lines HepG2, HuH6 and JHH5-A2, whilst showing no response to primary normal hepatocytes or primary cells from other organ systems. Greater increases in TCR affinity led to off-target activity, demonstrated as responses to primary normal target cells as well as antigen positive cell lines. This data has led to selection of a lead candidate for full pre-clinical assessment that demonstrates vastly improved efficacy against hepatocellular carcinoma cell lines, yet retains specificity by absence of response to primary normal tissue. This TCR may be suitable for further clinical studies in hepatocellular carcinoma.

